# Gene set internal coherence in the context of functional profiling

**DOI:** 10.1186/1471-2164-10-197

**Published:** 2009-04-27

**Authors:** David Montaner, Pablo Minguez, Fátima Al-Shahrour, Joaquín Dopazo

**Affiliations:** 1 Department of Bioinformatics and Genomics, Centro de Investigación Príncipe Felipe (CIPF), Valencia, E-46013, Spain; 2 Functional Genomics Node (INB), Centro de Investigación Príncipe Felipe (CIPF), Valencia, E-46013, Spain; 3 CIBER de Enfermedades Raras (CIBERER), Valencia, E-46013 Spain

## Abstract

**Background:**

Functional profiling methods have been extensively used in the context of high-throughput experiments and, in particular, in microarray data analysis. Such methods use available biological information to define different types of functional gene modules (e.g. gene ontology -GO-, KEGG pathways, etc.) whose representation in a pre-defined list of genes is further studied. In the most popular type of microarray experimental designs (e.g. up- or down-regulated genes, clusters of co-expressing genes, etc.) or in other genomic experiments (e.g. Chip-on-chip, epigenomics, etc.) these lists are composed by genes with a high degree of co-expression. Therefore, an implicit assumption in the application of functional profiling methods within this context is that the genes corresponding to the modules tested are effectively defining sets of co-expressing genes. Nevertheless not all the functional modules are biologically coherent entities in terms of co-expression, which will eventually hinder its detection with conventional methods of functional enrichment.

**Results:**

Using a large collection of microarray data we have carried out a detailed survey of internal correlation in GO terms and KEGG pathways, providing a coherence index to be used for measuring functional module co-regulation. An unexpected low level of internal correlation was found among the modules studied. Only around 30% of the modules defined by GO terms and 57% of the modules defined by KEGG pathways display an internal correlation higher than the expected by chance.

This information on the internal correlation of the genes within the functional modules can be used in the context of a logistic regression model in a simple way to improve their detection in gene expression experiments.

**Conclusion:**

For the first time, an exhaustive study on the internal co-expression of the most popular functional categories has been carried out. Interestingly, the real level of coexpression within many of them is lower than expected (or even inexistent), which will preclude its detection by means of most conventional functional profiling methods. If the gene-to-function correlation information is used in functional profiling methods, the results obtained improve the ones obtained by conventional enrichment methods.

## Background

The popularisation of high-throughput technologies such as DNA microarrays has lead to a parallel demand of methods for data analysis. In particular, the necessity of providing a functional interpretation at molecular level that accounts for the macroscopic observations in high-throughput experiments has promoted the development of different methods for the functional profiling of this type of experiments during the last years [1,2].

It is widely accepted that genes do not operate alone within the cell, but they carry out their functions through a complex interplay whose most obvious experimental evidence is the intricate network of protein interactions that we only just have started to decipher [3,4]. Most of the biological functionality of the cell arises from complex interactions between their molecular components that define operational interacting entities or modules [5]. Functions collectively performed by such modules can conceptually be represented in different ways, being possibly Gene ontology (GO) [6] and KEGG pathways [7] the most popular and widely used ones. For practical purposes, functional modules are defined as sets of genes sharing GO or KEGG annotations. There are, obviously, many other categorizations of gene modules in different domains; for example Reactome pathways [8], Biocarta pathways , etc.

In an attempt to understand the functional basis of high-throughput experimental results different functional profiling methods have been proposed [1]. Depending on the way the experimental data are selected and used two main families of methods, generically known as functional enrichment methods and gene set methods, can be distinguished. Functional enrichment methods have been implemented in several programmes such as GOMiner [9], FatiGO [10] and others. These are used to study whether a previously selected list of genes of interest is significantly enriched in one or more functional modules. Typical criteria for the selection of such gene lists in microarray experiments are differential expression between two classes, co-expression across experiments, etc. Thus, by means of this simple two-step approach, a reasonable biological interpretation of a microarray experiment can be achieved. Gene set methods were proposed more recently and directly aim to detect sets of functionally related genes (modules) with a coordinate and significant over- or under-expression across a list of ranked genes. Gene lists are ranked by differential expression between two classes, compared in microarray experiments [11-16]. In that way, the first step, in which genes are selected according to thresholds that ignore its cooperative behaviour, was avoided.

However, all these methods use functional modules as categorical variables. This fact, in the most typical microarray experimental designs (e.g. up- or down-regulated genes, clusters of co-expressing genes, etc.) or in other types of genomic experiments (e.g. Chip-on-chip, epigenomics, etc.), leads to the implicit assumption that such functional modules must be composed by sets of genes with a strong level of co-expression (otherwise they would never appear together in clustering or differential expression experiments or co-regulated by transcription factors, etc.). Nevertheless this assumption might not be necessarily true for all these modules. In fact, early attempts to deduce gene functionality (that is, functional module membership) from gene co-expression revealed that many functional modules did not even show a detectable degree of internal co-expression [17,18]. Therefore, if a non-negligible number of functional modules cannot be considered to be discrete categories there are two potential problems that affect all the methods for functional profiling: There is, on one hand, a problem of power in the statistical tests used, given that a number of functional modules tested will never be found simultaneously activated or deactivated, but are taken into account in the p-value adjustment procedures. On the other hand there is a potential problem of sensitivity, because many functional modules include genes with different degrees of intra-module co-expression, while the methods are many times applied to datasets in which the complete module is assumed to be over- or under-expressed as a whole. Since functional profiling methods do really produce results, one may conjecture that the results that are being obtained under the present unrealistic assumptions are only an underestimation of the results that could be really obtained if functional classes were properly tested.

Surprisingly, there are no systematic studies on the extent of this lack of internal co-expression within the most commonly used functional module definitions. The aim of this study was to produce a detailed survey on the GO and KEGG functional module definitions so as to determine which ones among them can be considered coherent modules of co-expression across a wide range of representative human samples. In principle, such coherent functional modules will define the subset of GO and KEGG functional modules susceptible of being detected using common strategies for functional profiling. In order to do so, we have derived, for each functional module as defined in GO and in KEGG, a co-expression (or coherence) index which could be used to assess the strength of its internal correlation. This index has been further used to filter for functional modules with a weak internal degree of co-expression. The index was derived from a gene pairwise correlation matrix representing the overall correlation structure of the human transcriptome as estimated from microarray expression measurements of 3034 samples collected under the most diverse biological conditions. In addition, a second main aim of this work was to use this information to re-define the functional modules as non-discrete entities. Even in the case of the functional modules with a high degree of internal coherence, these cannot be considered as co-expression modules but rather as entities with a core of co-expressed genes along with a variable number of genes with lower correlation (that probably modulate, complement or provide alternative functionality). In other words, not all the genes need to be expressed at the same time for the function to be activated. Then, for each gene annotated within a functional module, we estimate its degree of correlation with the main bulk of genes annotated under such module. In this way we provide an index which is useful for quantifying how essential each gene is in the activation of the functional module. At the same time, we introduce a framework within which functional modules can be treated as non-discrete entities. Under the prism of this new vision of gene function, we propose a simple modification of the functional profiling methodologies in order to enhance the use of biologically relevant information as described in the functional modules. Finally, we present some examples about how these modifications can enhance the detection of biologically meaningful functions which would have remained unnoticed using currently available techniques for functional enrichment.

## Results

### Coherence index applied to functional modules defined by GO and KEGG annotations

A coherence index that gives an idea of the internal correlation of the genes belonging to a functional module has been proposed and estimated for all the GO terms and KEGG pathways. This coherence index may have several interpretations but certainly the most direct one is its understanding as the complement of a p-value. We firstly calculate the all-against-all correlation matrix for all the 10866 transcripts across the 3034 arrays used (see material an methods section), which is available as online supplementary material . When the median correlation between the transcripts of a functional module is compared to the empirical distribution of correlations, estimated over randomly sampled sets of genes, we are assessing how strong the departure of our estimate from the null hypothesis of module correlation is. In other words, we can test if the internal correlation of such module is significantly higher than the correlation observed in a similar number of functionally unrelated genes. The coherence index proposed is the percentile represented by the module correlation within the random distribution. This index accounts for the complement of the probability of observing, under the null hypothesis, a value as extreme as the observed median. The cut-off of 0.05 usually chosen to reject a null hypothesis when the observed p-value is lower would be represented, in this case, by the level 95 of our coherence index. We would reject the null hypothesis for a functional module when its estimated index is higher than such value.

**Figure 1:**
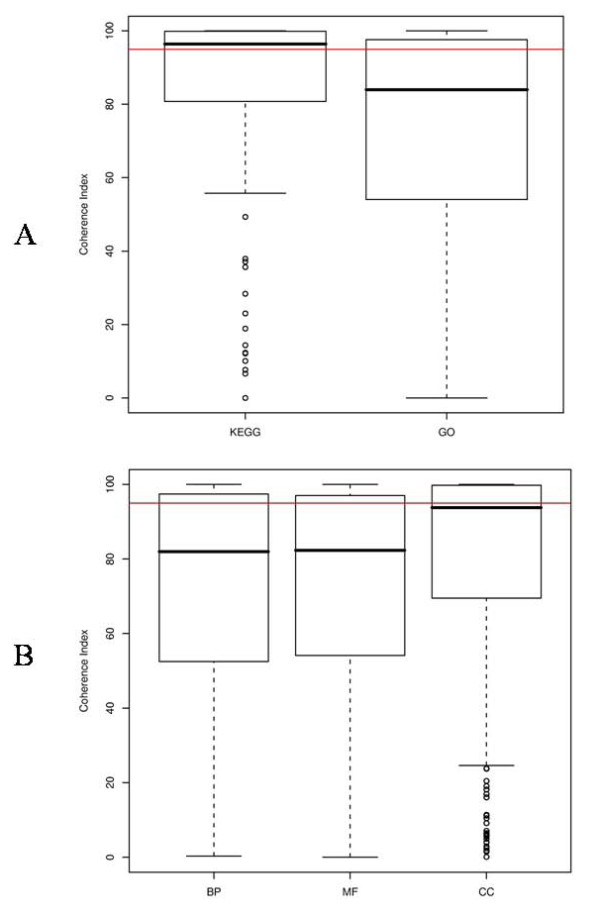
**Distribution of coherence indexes**. Coherence indexes for **A) **KEGG pathways and GO and **B) **the three GO Ontologies.

The application of the coherence index to the functional modules as defined by KEGG pathways showed that only 57% of them presented a correlation index greater than 95 (see Figure 1A). That is, if we were performing statistical analysis searching for KEGG pathways having internal correlation stronger than the overall correlation of the transcriptome, we would find no evidence of significant strong internal correlation in 43% of the cases. Thus 43% of the KEGG pathways do not co-express more than they would do if they were composed of functionally unrelated genes. Supplementary Dataset S1 contains the list of the KEGG pathways with their corresponding coherence index and median correlation values. Even more drastic are the results obtained for the GO terms. Only 32% (30% in Biological Process; 30% in Molecular Function; 46% in Cellular Component) of the functional modules defined by GO showed a correlation index greater than 95 (see Figure 1B). Supplementary Datasets S2, S3 and S4 contain the list of the GO terms corresponding to the "biological process", "molecular function" and "cellular component" ontologies respectively, along with their corresponding coherence indexes and median correlation values.

It is also worth pointing out that for many functional modules correlation indexes below 50 were observed. This means that for those modules the internal correlation is even lower than the overall genome correlation, which suggests the existence of a pattern of negative correlations among a significant amount of genes in the modules.

**Figure 2:**
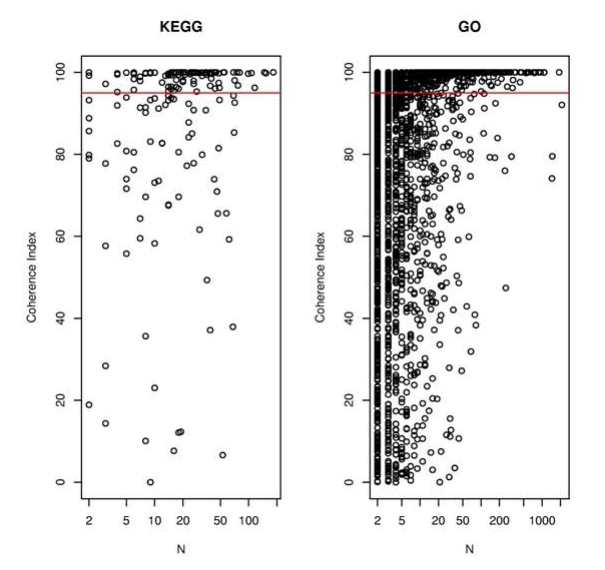
**Coherence index values as a function of functional module size obtained for KEGG (left) and GO (right) categories**.

As expected, large functional modules (more than 100 transcripts) tend to have a strong internal correlation whereas small modules show more variability (see Figure 2). This was also observed for the three ontologies (Biological Process, Molecular Function and Cellular Component) of GO (see Additional file 1)

Not surprisingly it was found that, in general, when the internal median correlation of a functional module was low (correlation index below 50) its estimated standard deviation was high (see Additional file 2). More interesting is the finding that many functional modules with high internal correlation had also high standard deviations. This last observation makes it clear that, even within the functional modules which have a strong internal co-expression, there exist a non-negligible number of genes which do not co-express with the main bulk of genes of the module. We may conclude that, while a number of GO terms or KEGG pathways are defining true functional modules of genes which need to be co-ordinately expressed in order to activate their corresponding functional roles, most of the currently used functional modules are not formed by sets of co-expressing genes.

### Coherence index and the level of annotation in GO

**Figure 3:**
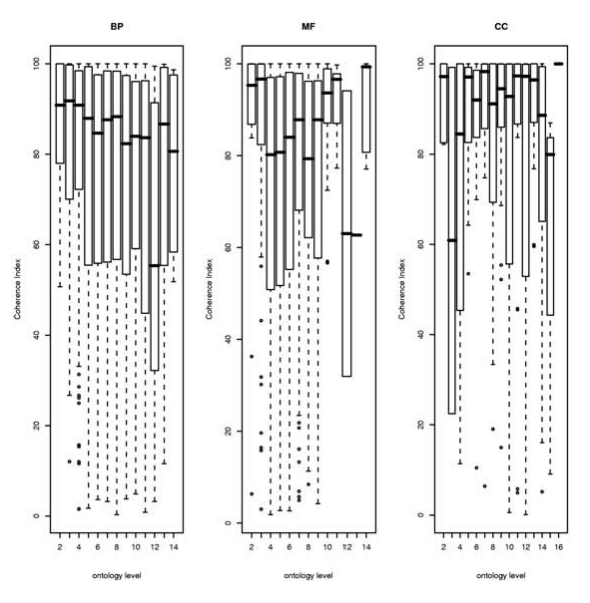
**Coherence index as a function of the level (the deeper the more specific the functional definition) in the GO hierarchy obtained for the three ontologies: Biological process (left), molecular function (center) and cellular component (right)**.

In the particular case of GO, where functional terms are related to each other following a special type of hierarchical structure called directed acyclic graph (DAG) [6], we have studied the relationship between the proposed coherence index and the level of annotation of each term. Here, the level of annotation of a GO term is defined as the maximum number of nodes that can be found in the DAG between the term and the root of the corresponding ontology. Under this definition, high levels in the ontology represent more specific GO terms. Our findings show (see Figure 3) that there is not a direct relationship between the degree of internal correlation of a GO term and its level in the ontology hierarchy. It is interesting to remark that, contrarily to what it was expected, more specificity in a GO term does not imply a tighter co-expression. This is probably a reflection of the fact that many definitions in the ontology are not accounting for simple cooperative processes such as the ones carried out for example, by a complex of proteins.

### Using gene-to-function information to best detect functional modules

In the following examples we show how to use this gene-to-function inter-dependence in order to incorporate the non-discrete nature of the membership of a gene to a functional module in the context of functional enrichment analysis.

#### Case example 1: functional profiling of genes differentially expressed in patients infected with Human Papillomavirus

A study of 36 Head and Neck Squamous Cell Carcinoma (HNSCC) tumour samples, 8 of them corresponding to patients infected with Human Papillomavirus (HPV+) and the remaining 28 to non infected patients (HPV-) [19] was used to illustrate the application of the proposed methodology. The authors assessed differential gene expression between HPV+ and HPV- tumours using Affymetrix 133 Plus 2.0 chips, and reported 89 genes over-expressed in the HPV+ group of tumours. A significant number of such genes were cell cycle regulators and transcription factors. The Affymetrix IDs for these 89 genes can be found in the supplementary material provided by the authors. The gene expression data are available in the GEO database [20] under the accession number GSE3292. In this case, raw files (.CEL) were not available and consequently these were not used in our estimation of the correlation between genes. Therefore, weights used in the analysis were obtained independently from the analyzed data set. The internal correlation value of any transcript to the rest of the transcripts in a functional module is used to assign a weight to it (see material and methods). Transcripts positively correlated to the rest of the module are given a weight of 2 (that is, are given double importance in the calculations), while negative correlations are penalised with a weight of 0.5 (half of the importance in this case). For the rest of genes a weight of 1 is used. A logistic regression model, which allows the use of weights, is utilised here instead the classical Fisher's test of equivalents (see material and methods).

We have systematically explored the GO and KEGG functional annotations of these 89 genes over-expressed in HPV+, testing for differences against the whole genome, that is, the remaining genes represented in the Affymetrix chip. A total 733 GO Biological Process terms and 161 KEGG pathways (with sizes comprised between 10 and 500 genes) were tested as described in the methods section.

A total of four GO terms were found as significantly over-represented in the group of over-expressed genes by the application of a standard, un-weighted test for functional enrichment with the permutation correction (see Table 1). In agreement with the discussion of the authors on the functionality of the genes differentially expressed [19], the terms related to DNA metabolism/replication (*DNA replication initiation*, p < 0.001, and *DNA strand elongation*, p < 0.001) were found. Also *SRP-dependent cotranslational protein targeting to membrane *(p < 0.001), probably accounting for the production of viral proteins is found. Finally, a term with no clear interpretation, *regulation of smooth muscle contraction*, was also found.

**Table 1:** Gene ontology functional terms and their respective significances under the standard (unweighted) and the weighted tests obtained for the HPV experiment [19] with the permutation test.

			**Weighted**			**Unweighted**		
**GO name**	**BP**	**size**	**Log Odds**	**p-value**	**Adjusted ** **p-value**	**Log Odds**	**p-value**	**Adjusted ** **p-value**

negative regulation of protein kinase activity	**GO:0006469**	100	3.030	<0.001	<0.001	2.548	0.006	0.083

DNA replication initiation	**GO:0006270**	44	4.281	<0.001	<0.001	4.162	<0.001	<0.001

SRP-dependent cotranslational protein targeting to membrane	**GO:0006614**	12	4.103	<0.001	<0.001	4.032	<0.001	<0.001

DNA strand elongation	**GO:0006271**	13	4.079	<0.001	<0.001	3.945	<0.001	<0.001

regulation of smooth muscle contraction	**GO:0006940**	18	2.875	0.010	0.088	3.597	<0.001	<0.001

The application of the alternative weighted analysis proposed here detects a new term: *negative regulation of protein kinase activity *(p < 0.001), while *regulation of smooth muscle contraction *disappears. It is long known the relationship between MAP kinase and growth factor activity, two terms descendant of *negative regulation of protein kinase activity *and HPV infection [21] (see Table 1).

In the equivalent analysis of functional modules defined using KEGG, the pathway Heparan sulfate biosynthesis (that remained unnoticed in the unweighted test) was found to be significantly over-represented in the genes over-expressed in HPV+ by the weighted test significant. It has recently been reported that Human Papillomavirus infection requires cell surface heparan sulfate [22]. *Urea cycle and metabolism of amino groups *is significant in both the weighted and the unweighted analysis.

#### Case Example 2: functional differences between two types of cancers

A second example on a matched-pair analysis of 24 breast tumours to study the transition between *in situ *ductal carcinoma (DCIS) and invasive ductal carcinoma (IDC) [23] was analysed. In the study Affymetrix HG U133A and HG U133 Plus 2.0 chips were used to assess gene expression differences between these two conditions. The authors reported 445 Affymetix probe-sets up-regulated in IDC and 101 down-regulated in IDC. In their analysis authors also indicate cell-to-cell signalling and interaction as being the more significant functions of the differentially expressed genes. As in the previous example, Affymetrix IDs of the differentially expressed probe-sets where provided and gene expression data are available in the GEO database [20] under the accession number GSE3893.

We have tested for enrichment in GO and KEGG terms in the up-regulated genes and in the down-regulated genes. A total of 733 GO terms of Biological Process and 161 KEGG pathways of sizes comprised between 10 and 500 genes where included in this study.

Using a standard, un-weighted test for functional enrichment with the permutation correction two KEGG pathways:, *Focal adhesion *(p < 0.001) and *ECM-receptor interaction *(p < 0.0001), as well as two GO terms: *transmembrane receptor protein tyrosine kinase signaling pathway *(p < 0.001) and *regulation of cell shape *(p < 0.001), all of them related with the maintenance of cellular structures and cell motility, were found as differentially expressed. Again, the application of the alternative weighted analysis proposed here detects a new term: *proteoglycan metabolism *(p < 0.001). Proteoglycans are known to determine mitogenic responses of breast carcinoma cells to fibroblast growth factors, mediated by tyrosine kinase-signaling receptors [24].

## Discussion

Functional annotations, such as GO or KEGG pathways, have been used for the definition of modules of genes in functional enrichment methods [1,9,10]. The detection of such functional modules within lists of genes by means of different tests relies upon the implicit assumption that common functionality implies a high degree of co-expression among all the members of each module [25]. While this assumption can be considered true as a general observation, it does not necessarily imply that the conventional definitions of functional classes used for this purpose (GO, KEGG, etc.) do all correspond to co-expressing sets of genes. It was previously reported that a large number of functional modules showed a low degree of internal co-expression, contradicting thus the expected cooperation among the genes to carry out their functions together [17,18]. Despite this observation, a systematic study on the degree of internal co-expression of the most commonly used functional modules and the impact of this bias on real biological data has not been carried out to date. Here we aimed a redefinition of functional modules, understood as groups of genes carrying out, cooperatively, a function in the cell. It is widely recognized that the biological circumstance of coexpression of two genes is properly defined by the coefficient of correlation among them [26]. So, we use it here to measure gene coordinate activity within a functional module. In this paper we present a general methodology to quantify the strength of the internal correlation of a functional module and we propose a simple way of using this information for functional profiling purposes that allows finding functional modules activated or deactivated that would remain otherwise unnoticed.

We have derived the correlation structure of the largest possible fraction of the human transcriptome, estimating its parameters from measurements from 3034 DNA microarrays stored in public data repositories. One of the strengths of the present study is, precisely, the big sample size (especially large if the difficulties in finding comparable microarrays in the databases are considered [27]) on which all estimations relay on. Of not less importance is the wide range of biological conditions considered in the study which includes several types of normal tissues, different kinds of cancer cells, male and female individuals as well as different cell lines. In order to ensure as much as possible the compatibility of the data gathered for the analysis, we have used one of the more extensively used expression arrays currently available (Affymetrix HG U133 Plus 2.0). For the same reasons, we have only collected datasets for which raw data were available so we could normalize and pre-process all of them together with the same method. This collection of samples constitutes a large dataset that allows us to perform a robust profiling of a large fraction of the human transcriptome, covering an ample spectrum of clinically and biologically relevant conditions.

The correlation structure of the transcriptome has been used to derive a coherence score which measures the internal co-expression of 173 KEGG pathways and 2221 GO terms. Our estimations indicate that only 57% of the KEGG pathways and just 32% of the GO terms can be considered to have internal correlation stronger than random modules of functionally unrelated genes of the same size. We also provided separate estimates for each of the Gene Ontologies (30% in BP; 30% in MF; 46% in CC), showing that, in general, GO Biological Processes or Molecular Function have a weaker internal correlation than KEGG pathways or GO Cellular Component. Another interesting finding was the fact that many modules have high internal correlation but also high variability.

Different reasons may account for these observations. In some cases there are functional modules defined in GO that are composed by independent or even antagonistic sub-modules and, consequently, their genes will never be found co-expressing in any experiment. Examples are transporters, which are composed by different independent types of sub-modules or any GO term starting by "regulation of", which usually has two antagonistic descendants called "positive regulation of" and "negative regulation of". In other cases, there are functional modules that require of a core of genes for properly carrying out the function and other genes of the module are only activated under particular physiological conditions, stresses, etc., displaying a lower degree of correlation. Modules composed by sub-modules can also exist, and many other situations can be imagined. In any case, the vision of a functional module as a discrete class, to which genes belong or do not belong to, is definitively not supported by the observations made. Thus, it is urgent to take a new approach that accounts for the non-discrete nature of the functional modules as defined by the most commonly used functional annotations (GO and KEGG).

In addition we highlighted how the level of annotation of a GO term in the ontology structure may not be the most suitable indicator, at least in terms of co-regulation, of the described function, despite being often used as a measure of its specificity.

Under the above mentioned considerations, most currently used functional profiling methods which model functional modules as groups of co-expressing genes, seem clearly inappropriate. The need of new methodologies for functional profiling and, above all, the essential requirement of a new notion of membership of a gene to a functional module is still an open issue. The proposed coherence score can be used in a first instance as a filtering criterion when the aim is to relate functionality to gene expression by discarding functional modules that will never be found as co-expressing units. Beyond this obvious use, this index can also be used to derive a weighting scheme that introduces the idea of non-discrete functional modules within the context of functional profiling methodologies in a straightforward manner. The proposed weighting scheme has the desirable property of using information on gene coexpression in the algorithm when such information is available but not introducing any bias when the information is missing. Relying on this new concept and using gene expression correlation information, we have shown with two examples how the proposed weighted approach discovers GO terms and pathways unnoticed under the equivalent standard un-weighted functional profiling method.

The approach shown here is quite general and could easily be extended to any other species or different platforms just by calculating the corresponding correlation matrix in a straightforward manner. The methodology could also be easily extended to any other types of modules defined by functionality, regulatory motifs, etc. Obviously, the use of newer strategies for functional profiling such as the different versions of gene set enrichment analysis [11-14,16], would benefit of considering this weighted definition of functional modules instead of using the classical categorical, un-weighted definitions.

Although the weighting schema proposed is quite simple, it proves efficient in finding functional modules in a standard functional enrichment analysis framework [1,10], as shown by the examples. Obviously, these examples have only an illustrative purpose of the application of the method that uses information on gene coexpression to improve functional module detection. However, in the worst scenario in absence of such information, this approach would be strictly equivalent to a conventional functional enrichment test and, therefore, its application would be equally valid. The use of most sophisticated weighting schemes, in which the continuous distribution of values of co-expression of all the genes in the module (and possibly outside the module) were taken into account, would probably improve even more the results. Also, a similar philosophy could also be applied to improve the detection of modules in gene-set enrichment methods although it falls beyond the scope of this manuscript.

## Conclusion

The aim of the manuscript was, on one hand to show the discrepancy between functional modules as defined in some popular repositories (GO and KEGG pathways) and real co-expressing modules and, on the other hand, to propose a new vision of such modules that combines the original definition of the function with the actual dynamics of co-expression. In this more realistic scenario, functional modules with a coherence index that makes them undistinguishable from functionally unrelated gene modules would be excluded from a functional analysis, thus increasing the power of any test in the process of adjustment for multiple testing. In the remaining functional modules to be tested, more importance will be given to the core of co-expressing genes while uncorrelated genes and negatively correlated genes (probably representing genes that express under particular physiological conditions or stress situations, or perhaps other sub-modules with an independent dynamics of expression) will be penalised in the analysis.

Despite functional profiling of genome-scale experiments is an active field in which new proposals arise continuously [1,2], the concept of functional modules as binary discrete classes has remained unchanged along the last years. With the coherence index and the weighted schema proposed here we have introduced a conceptually new operative definition of functional module, biologically more meaningful, that clearly increases the sensitivity of functional profiling methods.

## Methods

### Expression values

All data used in this study was downloaded from the Gene Expression Omnibus (GEO), public repository of the NCBI [20]. At the time of doing this study, there were 169 GEO "series" containing microarray data generated using the Affymetrix GeneChip Human Genome U133 Plus 2.0 Array (GPL570 platform in the GEO data base). Only for 74 of those series raw data (Affymetrix .CEL files) were available, comprising a total of 3034 array hybridized to all kind of human samples. We downloaded the raw data (.CEL files) for the 3034 arrays, normalized them in batches of size 100 (because of memory size limitations) using the function RMA in the *affy *library of Bioconductor [28] and finally rescaled all batches together using the "quantile" method implemented in the *limma *library of Bioconductor [29].

The data covered an ample spectrum of biological conditions including different tissues, and diseases, male and female individuals as well as cell lines.

### ID mapping

Affymetrix probe-set identifiers were linked to their corresponding transcripts according to the Ensembl database, release 44 [30]. Among the 54675 probe-set IDs in the Affymetrix chip just 31542 had a corresponding Ensembl Transcript ID. Such IDs where unique just for 15477 Affymetrix IDs; that is, there are 16065 of the Affymetrix IDs that correspond to at least two different Ensembl Transcripts. A requirement of this study was to generate transcript expression measurements independent one of each other. Therefore we used just the 15477 Affymetrix IDs mapping to unique Ensembl IDs and, when several of them mapped to the same transcript, summarize them by its mean. In this way we manage to compute expression levels for 10866 transcripts, corresponding to 10486 genes of 3034 human samples.

### Definition of functional modules using GO and KEGG annotations

GO and KEGG pathways annotation for the Affymetrix HG U133 Plus 2.0 array (the most abundant microarray in the databases) was taken from the Bioconductor meta-data package "hgu133plus2" (version 1.14.0, see ) which is assembled using data from public data repositories. 2221 GO terms (1014 Biological Process; 925 Molecular Function; 282 Cellular Component, Built: 8-Aug-2006) and 173 KEGG pathways (Release 38.1, June 1, 2006) that had annotated at least two of the 10866 selected transcripts where used in this study. While KEGG pathways are conceptually considered as independent entities, GO terms are related among them by a hierarchical relationship (known as directed acyclic graph, or DAG, in which a term can have more than one parent). Terms closer to the root define more general concepts and terms towards the leaves define more specific terms. In the particular case of GO terms, the usual procedure is to consider that each gene annotated to a given level is automatically annotated to all its parents [1]. All the GO terms have been used without making any distinction among distinct evidence codes. Since an overwhelming majority are electronic annotations (IEA), neither here, not in the most common programs for functional profiling [1] are taken into account. Functional modules are therefore defined as sets of genes sharing GO or KEGG annotations.

### Computing correlations and assessing their strength

**Figure 4:**
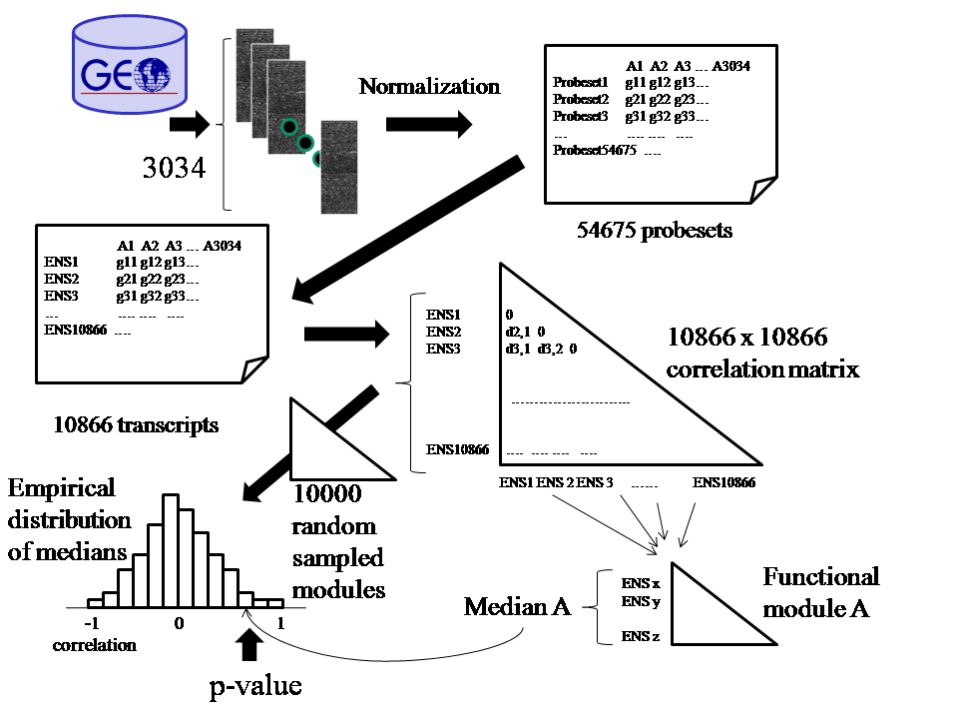
**Schematic representation of the procedure followed for obtaining the internal correlation for each functional module and its significance**. See material and methods.

The main motivation in this work is the redefinition of the essence of a functional module, understood as a group of genes carrying out, cooperatively, a function in the cell. Typically, the coefficient of correlation [26], which accounts for the coexpression of genes across the experimental conditions measured, is used to measure such gene cooperation within a functional module. Figure 4 illustrates the way in which we proceed for computing the internal correlations for all the functional modules and estimating its significance. Thus, for all pairs of transcripts, the correlation of their expression levels along the 3034 arrays was computed and stored in a 10866 by 10866 correlation matrix. Distribution of this correlation coefficients within the functional modules considered in this study (GO terms and KEGG pathways) was studied and summarized by a median correlation value for each of the terms. For each functional module (GO term or KEGG pathway) consisting of N transcripts we randomly sampled, from the whole collection of transcripts in the study, 10000 modules of the same size N. Then, for each of the 10000 resampled modules, we computed the median value of the correlation between its transcripts. In this way we obtained a sampling distribution of the median correlation within equivalent modules of transcripts of size N not functionally related. In order to assess how strong the real internal median correlation of each functional module is, any of these values was compared to the sampling distribution of median correlations of random (functionally unrelated) modules of the same size. The percentile of the sampling distribution represented by the true median correlation in the functional module is, finally, taken as a measurement of the strength of its internal correlation and provided as coherence index.

### The weighted approach: using co-expression information to improve functional profiling analysis

The most widely used tools for functional profiling classify genes into 2 by 2 contingency tables according to their functional annotation (functional module membership) and to the list to which they belong to. Then some statistical test, like a chi-square, Fisher or other equivalent test, is used to find statistically significant over-representations of any functional annotation in the lists of genes compared. Here we use logistic regression models [31] to estimate the log odds ratio of association between being or not annotated within a functional module and belonging to one or the other list of genes. When applied to binary data, this approach is equivalent to other 2 by 2 contingency table methods but has the advantage of allowing for the use of weighted observations. It has been shown that, when correlated genes are introduced in 2 by 2 contingency tables, standard tests inflate type I error rates [2]. In this paper we computed p-values based on the subject sampling model (1000 permutations) described by Goeman [2] in order to avoid such bias.

Here, we propose a very simple modification of the use of functional modules that can be applied within the context of functional enrichment analysis. The rationale for this modification is to give more importance to those genes that, being annotated in a functional module, are positively correlated to the main bulk of genes in the module. Likewise we seek to penalise the negative contribution to the detection of a functional module of those genes negatively correlated to this module. In order to achieve this, we have first to determine a measure of the internal correlation of genes within functional modules. Then, instead of using a discrete definition of functional modules, the correlations will be used to weight the membership of each gene to the module. When using the logistic model to test for each functional module, each gene was weighted depending on whether it was annotated or not within the module and whether it was positively or negatively correlated with it. Genes belonging to the functional module were given weight 2 if they were positively correlated to it and weighted by 0.5 if the correlation with the module was negative. The genes that were not in the functional module were given a neutral weight of 1. As in the classical functional enrichment test scenario, all computed p-values where corrected for multiple testing using the False Discovery Rate (FDR) method [32].

## Abbreviations

**DAG**: Directed acyclic graph; **FDR**: False Discovery Rate; **GEO**: Gene Expression Omnibus; **GO**: Gene Ontology; **KEGG**: Kioto Encyclopaedia of Genes and Genomes.

## Authors' contributions

DM is the author of the algorithm and has participated in all the analyses, FAS and PM has participated in the analysis of the data and JD has conceived and coordinated the study and written the manuscript. All the authors have read and approved the final manuscript.

## Supplementary Material

Additional File 1 **Additional Figure 1**. Coherence index values as a function of functional module size obtained for the three GO ontologies: Biological process (left), molecular function (center) and cellular component (right). Click here for file

Additional File 2 **Additional Figure 2**. Relationship between the coherence index and its standard deviation for KEGG (up left), GO biological process (up right), molecular function (down left) and cellular component (down right). Click here for file
