# Supplementary figures and images for: Gene set internal coherence in the context of functional profiling
Source: BMC Genomics. 2009 Apr 27;10:197. doi: 10.1186/1471-2164-10-197 (PMC2680416; doi:10.1186/1471-2164-10-197)

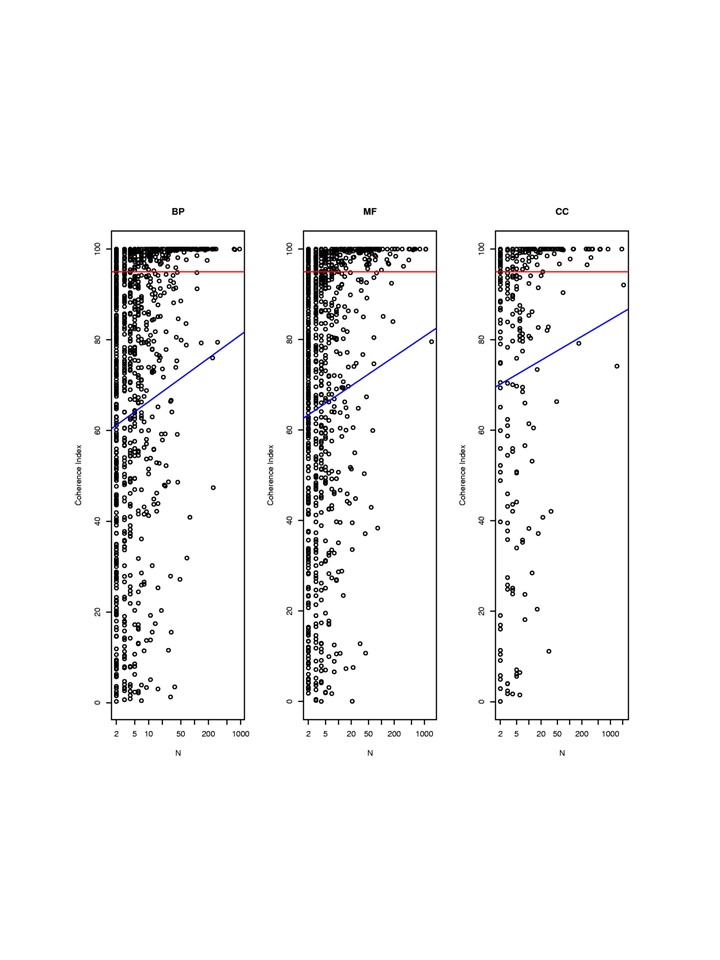

Supplement: Additional File 1 — Additional Figure 1. Coherence index values as a function of functional module size obtained for the three GO ontologies: Biological process (left), molecular function (center) and cellular component (right). [file 1471-2164-10-197-S1.jpeg]

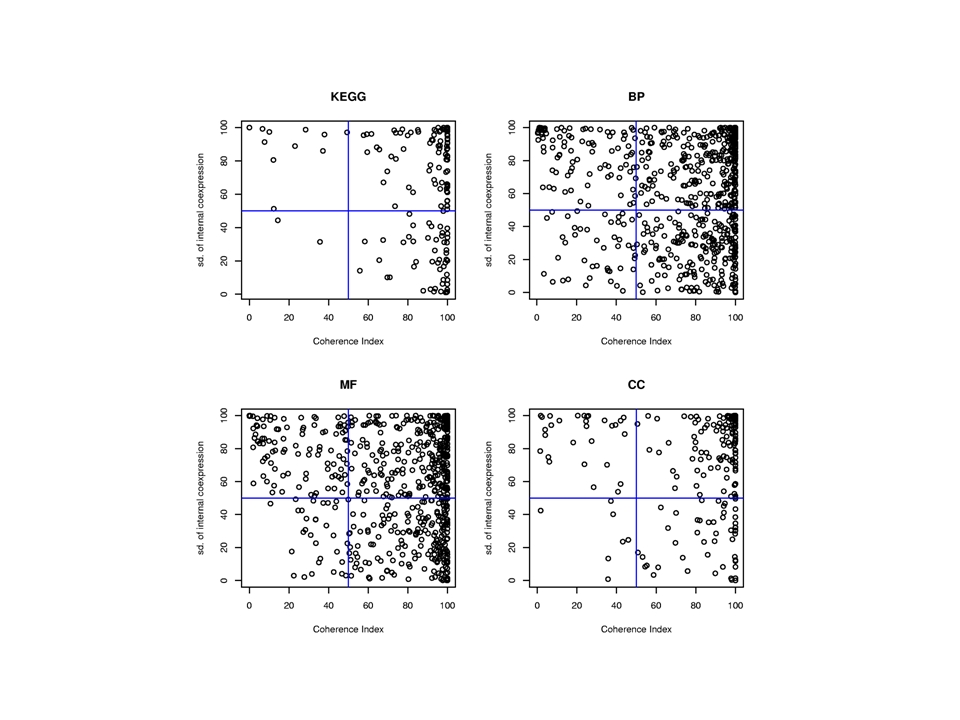

Supplement: Additional File 2 — Additional Figure 2. Relationship between the coherence index and its standard deviation for KEGG (up left), GO biological process (up right), molecular function (down left) and cellular component (down right). [file 1471-2164-10-197-S2.jpeg]
